# Omega-3 polyunsaturated fatty acid supplementation attenuates microglial-induced inflammation by inhibiting the HMGB1/TLR4/NF-κB pathway following experimental traumatic brain injury

**DOI:** 10.1186/s12974-017-0917-3

**Published:** 2017-07-24

**Authors:** Xiangrong Chen, Shukai Wu, Chunnuan Chen, Baoyuan Xie, Zhongning Fang, Weipeng Hu, Junyan Chen, Huangde Fu, Hefan He

**Affiliations:** 1 Department of Neurosurgery, the Second Affiliated Hospital, Fujian Medical University, Quanzhou, 362000 Fujian Province China; 2 Department of Neurology, the Second Affiliated Hospital, Fujian Medical University, Quanzhou, 362000 Fujian Province China; 3 Department of Neurosurgery, Affiliated Hospital of YouJiang Medical University for Nationalities, Baise, 533000 Guangxi Province China; 4 Department of Anesthesia, the Second Affiliated Hospital, Fujian Medical University, Quanzhou, 362000 Fujian Province China

**Keywords:** Traumatic brain injury, Omega-3 polyunsaturated fatty acid, Microglia, Neuroinflammation, HMGB1/TLR4/NF-κB pathway

## Abstract

**Background:**

Microglial activation and the subsequent inflammatory response in the central nervous system play important roles in secondary damage after traumatic brain injury (TBI). High-mobility group box 1 (HMGB1) protein, an important mediator in late inflammatory responses, interacts with transmembrane receptor for advanced glycation end products (RAGE) and toll-like receptors (TLRs) to activate downstream signaling pathways, such as the nuclear factor (NF)-κB signaling pathway, leading to a cascade amplification of inflammatory responses, which are related to neuronal damage after TBI. Omega-3 polyunsaturated fatty acid (ω-3 PUFA) is a commonly used clinical immunonutrient, which has antioxidative and anti-inflammatory effects. However, the effects of ω-3 PUFA on HMGB1 expression and HMGB1-mediated activation of the TLR4/NF-κB signaling pathway are not clear.

**Methods:**

The Feeney DM TBI model was adopted to induce brain injury in rats. Modified neurological severity scores, brain water content, and Nissl staining were employed to determine the neuroprotective effects of ω-3 PUFA supplementation. Assessment of microglial activation in lesioned sites and protein markers for proinflammatory, such as tumor necrosis factor (TNF)-α, interleukin (IL)-1β, IL-6, interferon (IFN)-γ, and HMGB1 were used to evaluate neuroinflammatory responses and anti-inflammation effects of ω-3 PUFA supplementation. Immunofluorescent staining and western blot analysis were used to detect HMGB1 nuclear translocation, secretion, and HMGB1-mediated activation of the TLR4/NF-κB signaling pathway to evaluate the effects of ω-3 PUFA supplementation and gain further insight into the mechanisms underlying the development of the neuroinflammatory response after TBI.

**Results:**

It was found that ω-3 PUFA supplementation inhibited TBI-induced microglial activation and expression of inflammatory factors (TNF-α, IL-1β, IL-6, and IFN-γ), reduced brain edema, decreased neuronal apoptosis, and improved neurological functions after TBI. We further demonstrated that ω-3 PUFA supplementation inhibited HMGB1 nuclear translocation and secretion and decreased expression of HMGB1 in neurons and microglia in the lesioned areas. Moreover, ω-3 PUFA supplementation inhibited microglial activation and the subsequent inflammatory response by regulating HMGB1 and the TLR4/NF-κB signaling pathway.

**Conclusions:**

The results of this study suggest that microglial activation and the subsequent neuroinflammatory response as well as the related HMGB1/TLR4/NF-κB signaling pathway play essential roles in secondary injury after TBI. Furthermore, ω-3 PUFA supplementation inhibited TBI-induced microglial activation and the subsequent inflammatory response by regulating HMGB1 nuclear translocation and secretion and also HMGB1-mediated activation of the TLR4/NF-κB signaling pathway, leading to neuroprotective effects.

## Background

Traumatic brain injury (TBI)-induced secondary injury is a complicated pathophysiological procedure that includes microglial activation, inflammatory cytokine release, oxidative stress, and abnormal mitochondrial activities, all of which inhibit functional repair after TBI [1–3]. TBI-induced microglial activation and the release of inflammatory factors, such as tumor necrosis factor (TNF), interleukin (IL), and interferon (IFN), cause direct neuronal cell death and also induce vascular endothelial cells to express a variety of cell adhesion molecules and cell chemotaxis. In addition, microglial activation can stimulate nitric oxide synthesis, which leads to increased capillary permeability, blood–brain barrier dysfunction, brain edema, and promotion of neuronal apoptosis [4, 5]. The inhibition of TBI-induced microglial activation and the subsequent neuroinflammatory response has been shown to improve the recovery in TBI patients [6].

High-mobility group box 1 (HMGB1), a member of the HMG family, is a non-histone-binding protein and is central to late inflammatory responses [7, 8]. Under normal physiological conditions, HMGB1 is located in the nucleus and binds with DNA to stabilize chromosomal structure and also regulate transcription and translation [8]. However, under pathophysiological conditions such as TBI and ischemia, cellular damage/death can induce HMGB1 translocation from the nucleus to the extracellular space, where it induces microglial activation causing further release of HMGB1 [9–11]. Extracellular HMGB1 binds to transmembrane receptors such as receptor for advanced glycation end products (RAGE) and toll-like receptors (TLRs) to induce further release of inflammatory cytokines [12, 13]. TLR4, a member of the toll-like receptor family, is an important transmembrane receptor involved in inflammation and plays an important role in HMGB1-mediated inflammatory processes [14]. Furthermore, TLR4 mediates other exogenous ligands, such as lipopolysaccharide (LPS), and also endogenous ligands, all of which elicit inflammatory responses that stimulate microglial activation and further release of inflammatory factors [15, 16]. HMGB1 activates TLR4 through both the medullary differentiation factor (MyD88) and non-MyD88-dependent pathways, triggering a signal cascade, either directly through the nuclear factor-κB (NF-κB) pathway or indirectly via the PI3K or MAPKs pathway. Following nuclear translocation of NF-κB, the expression of large numbers of inflammatory cytokines, as well as cascade amplification of inflammatory responses, occur [17–19]. Therefore, the investigation of the roles of HMGB1 translocation and release, and HMGB1-mediated activation of the TLR4/NF-κB signaling pathway in TBI-induced microglial activation and the subsequent inflammatory response, is paramount to further understand the mechanisms of TBI-induced secondary brain damage.

Omega-3 polyunsaturated fatty acid (ω-3 PUFA) plays a key role on human metabolism and includes α-linolenic acid, eicosapentaenoic acid, and docosahexaenoic acid [20]. Recent studies showed that ω-3 PUFA not only provides energy support through metabolism but also regulates inflammatory responses and immune function and also maintains internal organ functions [21, 22]. In addition, ω-3 PUFA affects the synthesis of lipids, regulates both the inflammatory response and release of inflammatory factors, and has antioxidative and anti-inflammation effects; all of which influence the pathogenesis of many diseases, including Alzheimer’s disease, multiple sclerosis, Parkinson’s disease, and cerebral ischemia [23–26]. Several studies have shown that ω-3 PUFA inhibited TBI-induced inflammatory responses and that this inhibitory mechanism may be related to microglial activation [27–29]. To date, no studies have elucidated if ω-3 PUFA affects HMGB1 translocation and release, or the exact mechanisms involved in HMGB1-mediated activation of the TLR4/NF-κB signaling pathway in TBI-induced microglial activation and the subsquent inflammatory response [15, 16]. In the present study, we investigated whether the immunonutrient, ω-3 PUFA, inhibited TBI-induced microglial activation and the subsequent inflammatory response and also facilitated neuronal recovery through inhibition of both HMGB1 translocation and release, and HMGB1-mediated activation of the TLR4/NF-κB signaling pathway.

## Methods

### Animals

All animal experiments were approved by the Fujian Provincial Medical University Experimental Animal Ethics Committee (Fuzhou, China) and were performed under strict supervision. Adult male Sprague–Dawley rats, ranging between 230 and 260 g, were purchased from the Experimental Animal Facility in Fujian Medical University and housed in a temperature (23 ± 2 °C) and light (12 h light/dark cycle)-controlled room with ad libitum access to food and water.

### Experimental model and drug administration

All rats were randomly assigned into a sham group, TBI group, and TBI + ω-3 PUFA supplementation group (TBI + ω-3 group) (*n* = 36 each). After injury, the groups were further divided into three subgroups: 1, 3, and 7 days. TBI was induced in anesthetized (50 mg/kg sodium pentobarbital; intraperitoneally) rats as described previously [30]. Briefly, a midline incision was made over the skull, and a 5-mm craniotomy was drilled through the skull 2 mm caudal to the left coronal suture and 2 mm from the mid line without disturbing the dura. TBI was induced using a weight-drop hitting device (ZH-ZYQ, Electronic Technology Development Co., Xuzhou, China) with a 4.5-mm-diameter cylinder bar weighing 40 g from a height of 20 cm. Bone wax was used to seal the hole, and the scalp was sutured. All procedures were the same for each group except in the sham group, in which no weight was dropped. Approximately 30 min after TBI, the TBI + ω-3 group was intraperitoneally injected with ω-3 PUFA (2 ml/kg; Sigma, St. Louis, MO, USA) once per day for seven consecutive days [23]. The remainder of the groups were injected with same dose of 0.9% NaCl as a control. As LPS is known to be a TLR4 agonist that activates TLR4 signaling to mediate inflammatory responses [31, 32], the rats were injected with 2 mg/kg LPS intraperitoneally 24 h after intraperitoneal ω-3 PUFA to clarify the role of TLR4/NF-κB in ω-3 PUFA neuroprotection.

### Assessment of neurological injury

Modified neurological severity scores (mNSS) [30] were used to evaluate the motor (muscle state, abnormal movement), sensory (visual, tactile, and proprioceptive), and reflex systems of rats. The mNSS test is graded on a scale of 0–18, where a total score of 18 points indicates severe neurological deficits and a score of 0 indicates normal performance; 13–18 points indicates severe injury, 7–12 indicates mean to moderate injury, and 1–6 indicates mild injury. Evaluation was performed 1, 3, and 7 days after TBI by investigators who were blinded to the experiments.

### Measurement of brain water content

After evaluation of neurological injury, the rats were sacrificed by decapitation and the brains were harvested immediately. A portion of the cerebral cortex (2 mm around craniotomy; 200 ± 20 mg) was isolated, and both blood and cerebrospinal fluid were removed using filter paper before wrapping the sample into pre-weighed aluminum foil. After wet weight was measured using a digital scale, samples were placed in an oven to dry for 24 h at 100 °C. Dry weight was measured again for each sample. Brain water content was found using the formula: % = 100% × (wet weight − dry weight)/dry weight.

### Nissl staining

Cortical tissue from lesion areas were fixed in formaldehyde, embedded in paraffin, and cut into 4-μm sections. Slices went through xylene dewaxing and an alcohol gradient rehydration as above and stained with Nissl solution (Boster Biotech, Wuhan, China) for 5 min. Compared to normal neurons, the cell bodies of injured neurons were shrunken and/or contained vacuoles and the nuclei stained darker. A pathologist who was blinded to the experiments randomly selected five random regions of interest (ROIs) under a high magnification optical microscope (×400; Leica, Wetzlar, Germany) to observe positively stained cells surrounding injury areas. Five random ROIs were selected for quantification, and the mean was used for the statistical analysis.

### Measurement of neuronal apoptosis by TUNEL staining

A TUNEL assay was performed using an apoptosis kit according to the manufacturer’s instructions (Roche Inc., Indianapolis, IN, USA). Sample slices were incubated with NeuN (1:100; Boster Biotech) overnight at 4 °C, and after three washings in PBS, the samples were incubated with TUNEL reaction mixture for 1 h at 37 °C. Double blinding was used for quantification. TUNEL-positive neurons surrounding the injury areas were observed and counted under high magnification (×400) on a ZEISS HB050 inverted microscope (Oberon Cohen, Germany). Five ROIs were selected for quantification, and the mean was used for the statistical analysis.

### Immunohistochemical and immunofluorescent staining

Cortical tissues from the lesioned areas were embedded in paraffin after being fixed in formaldehyde and cut into 4-μm sections. Slices went through xylene dewaxing, gradient alcohol hydration, and a citric acid buffer microwave protocol for antigen retrieval. For immunohistochemistry, the sections were incubated with antibodies against HMGB1 (1:100; Cell Signaling Technology, Danvers, MA, USA), Iba-1 (1:200; Santa Cruz Biotechnology, CA, USA), and GFAP (Abcam, Cambridge, UK) overnight at 4 °C. For immunofluorescence, the sections were incubated with antibodies against NeuN (1:100; Boster Biotech), Iba-1 (1:200; Santa Cruz), and HMGB1 (1:100; Cell Signaling Technology) overnight at 4 °C. Following washing, the sections were then incubated with secondary goat anti-rabbit IgG antibodies (Alexa Fluor 488 or Alexa Fluor 594, 1:200, Invitrogen, Grand Island, NY, USA) for an additional 1 h at room temperature. The cell nuclei were stained with 4′,6-diamidino-2-phenylindole (DAPI). A pathologist who was blinded to the experiments randomly selected five ROIs using a ZEISS HB050 inverted microscope system (×400; Oberon Cohen, Germany) to observe positively stained cells surrounding the injury areas. Evaluation of immunohistochemical sections was undertaken by assessing the intensity of staining (five grades) [33] where 0 indicated no detectable positively stained cells; 1 indicated a very low density of positively stained cells; 2 indicated a moderate density of positively stained cells; 3 indicated a higher, but not maximal density of positively stained cells; and 4 indicated the maximal density of positively stained cells. A mean of five ROIs was used for statistical analysis.

### Detection of inflammatory factors by enzyme-linked immunosorbent assay (ELISA)

Inflammatory factors in brain tissues were detected using an ELISA kit according to the manufacturer’s instructions (Biocalvin Company, Suzhou, China). Briefly, TNF-α, IL-1β, IL-6, IFN-γ, and HMGB1 (Boster Biotech) in both standards and samples were sequentially incubated with respective monoclonal antibodies and biotinylated anti-rat antibody, followed by horseradish peroxidase. Measured OD values were converted into a concentration value.

### Western blot analysis

Proteins were extracted with RIPA lysis buffer (Santa Cruz Biotechnology), and 30 μg of total protein was loaded on a gel and separated by sodium dodecyl sulfate-polyacrylamide gel electrophoresis. Proteins were transferred to polyvinylidene difluoride membranes and probed with primary antibodies against cleaved caspase-3 (1:300, Cell Signaling Technology), Bax (1:500, Abcam), SIRT1, Iba-1, phosphorylated (p)-IκB, TLR4, and NF-κB p65 (1:200, Cell Signaling Technology), followed by incubation with appropriate horseradish peroxidase-conjugated IgG (1:5000, Boster Biotech) secondary antibodies. Immunoblots were visualized using the Millipore ECL Western Blotting Detection System (Millipore, Billerica, MA, USA). Expression levels were normalized against β-actin (1:5000, Boster Biotech) or Lamin B1 (1:3000, Cell Signaling Technology).

### Statistical analysis

All statistical analyses were performed using SPSS 18.0 statistical software (SPSS Inc., Chicago, IL, USA). The results were expressed as mean ± standard deviation. Statistical differences among the groups were assessed by one-way ANOVA, and post hoc multiple comparisons were performed using Student-Newman-Keuls tests. Values of *p* < 0.05 were considered statistically significant.

## Results

### Neuroprotective effects of ω-3 PUFA supplementation after TBI

**Fig. 1:**
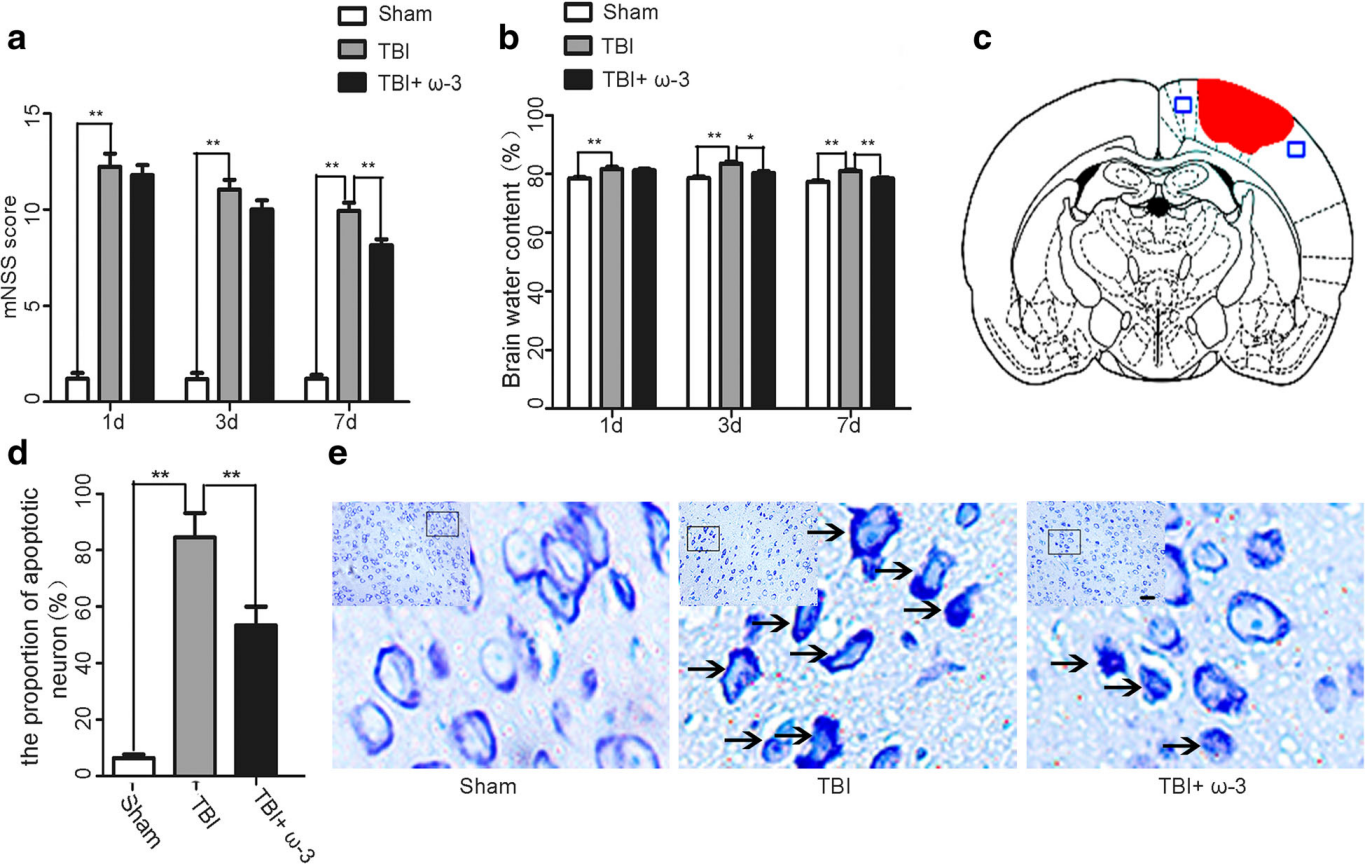
Effects of ω-3 PUFA supplementation on neurological function, brain edema, and neuronal apoptosis after TBI. **a** ω-3 PUFA supplementation improved neurological functions 7 days after TBI (8.14 ± 0.31 vs. 9.93 ± 0.42, *p* < 0.05). **b** ω-3 PUFA supplementation decreased brain water content 3 days after TBI (80.37 ± 0.54% vs. 83.62 ± 0.73%, *p* < 0.05). **c** A schematic of a brain section after TBI. Areas in *red* refer to the lesion sites, and areas in *blue* refer to the sample points, **d**, **e** ω-3 PUFA supplementation decreased neuronal apoptosis in lesioned cortices (53.47 ± 6.54% vs. 84.50 ± 8.69%, *p* < 0.05). Representative photomicrographs of Nissl staining in the experimental groups. *Arrows* point to apoptotic neurons; *n* = 6 in each group. The values are expressed as mean ± standard deviation: *p < 0.05, **p < 0.01, *scale bars* = 50 μm

Using mNSS to assess neurological functions in all groups [30], rats from the TBI group showed the highest levels of neurological deficits 1 day after TBI (12.23 ± 0.69). From day 7 after TBI, rats in the TBI + ω-3 group showed significantly better neurological functions than rats in the TBI groups (8.14 ± 0.31 vs. 9.93 ± 0.42, *p* < 0.05) (Fig. 1a).

Brain water content is a critical indicator used to evaluate prognosis after TBI [34]. Compared with the sham group, brain water content in the TBI group was significantly increased (*p* < 0.05). Supplementation with ω-3 PUFA significantly decreased brain water content 3 days after TBI compared to the TBI group (80.37 ± 0.54% vs. 83.62 ± 0.73%, *p* < 0.05) (Fig. 1b). Nissl staining was used to evaluate neuronal apoptosis in lesioned cortices [34]. Results demonstrated that the TBI group had significantly higher percentages of apoptotic cells in the cortex 3 days after TBI compared to the sham group (*p* < 0.05). However, compared to the TBI group, the TBI + ω-3 group had significantly lower percentages of apoptotic cells (53.47 ± 6.54% vs. 84.50 ± 8.69%, *p* < 0.05) (Fig.1c–e), suggesting ω-3 PUFA supplementation had neuroprotective effects after TBI.

### ω-3 PUFA supplementation inhibits microglia-mediated inflammatory response in the central nervous system (CNS)

**Fig. 2:**
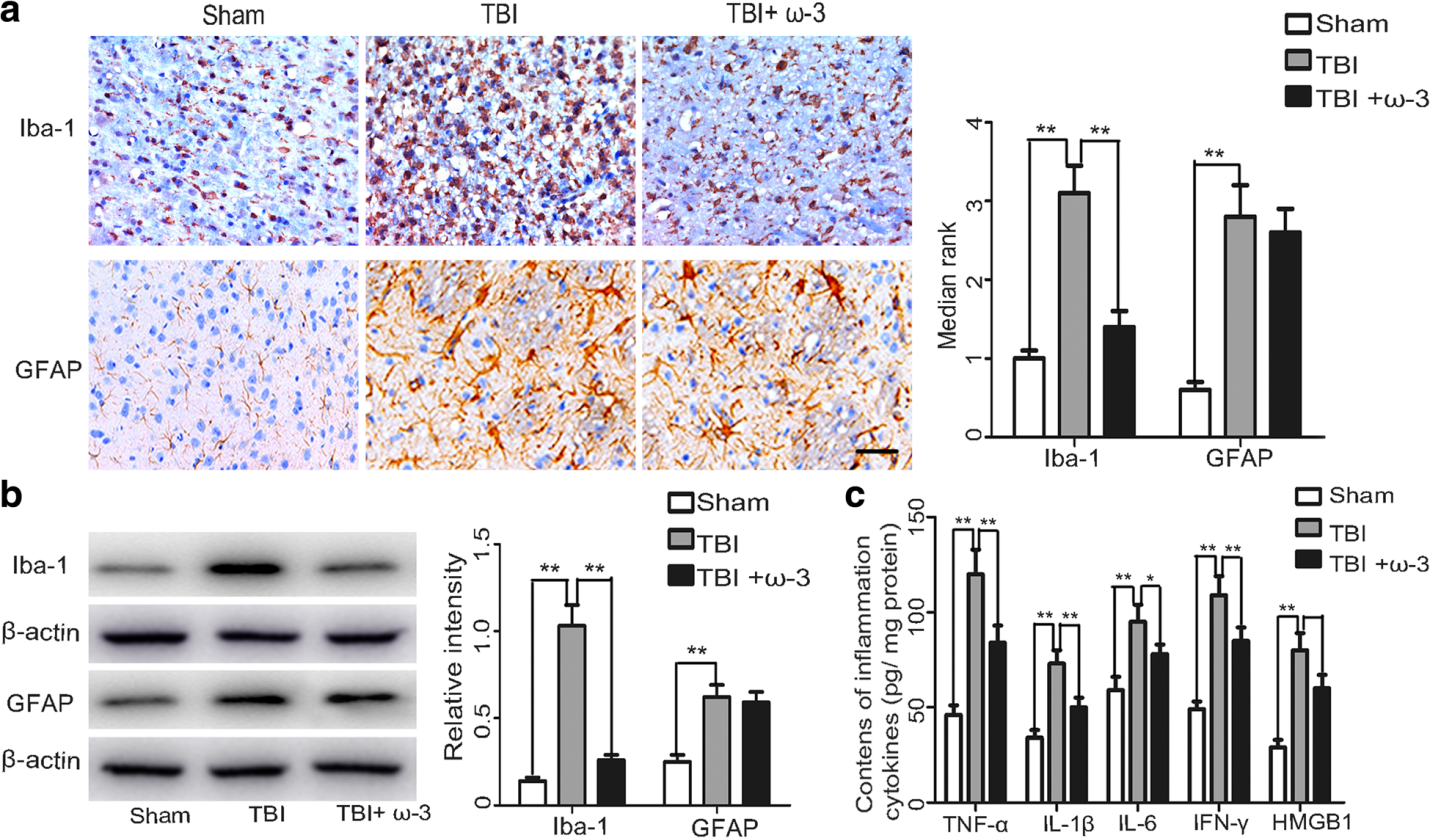
ω-3 PUFA supplementation inhibits microglial activation and the subsequent inflammatory response. **a** The microglial marker, Iba-1, was significantly decreased by ω-3 PUFA supplementation, but not the astrocytic marker, GFAP. **b** ω-3 PUFA supplementation decreased Iba-1 protein levels (1.44 ± 0.21 vs. 3.13 ± 0.35, *p* < 0.05), but not GFAP protein levels (2.62 ± 0.31 vs. 2.83 ± 0.42, *p* > 0.05). **c** ω-3 PUFA supplementation significantly decreased TBI-induced enhancement of TNF-α, IL-1β, IL-6, IFN-γ, and HMGB1 (*p* < 0.05); *n* = 6 in each group. The values are expressed as mean ± standard deviation: *p < 0.05, **p < 0.01, *scale bars* = 50 μm

Considering the important effects of microglial activation and the subsequent neuroinflammatory response on secondary damage after TBI [6, 35], the effect of ω-3 PUFA supplementation on microglial activation and expression of inflammatory factors 3 days after TBI was investigated. Both immunohistochemical staining and western blot analysis showed that activated microglial cells (Iba-1^+^) and astrocytes (GFAP^+^) were increased after TBI. Interestingly, ω-3 PUFA supplementation significantly inhibited microglial activation (1.44 ± 0.21 vs. 3.13 ± 0.35, *p* < 0.05), but not astrocyte activation (2.62 ± 0.31 vs. 2.83 ± 0.42, *p* > 0.05) (Fig. 2a, b). Expression levels of inflammatory factors (TNF-α, IL-1β, IL-6, IFN-γ, and HMGB1) were measured after TBI using an ELISA kit, and results showed that the TBI group had significantly higher expression levels of inflammatory factors compared to the sham group, while ω-3 PUFA supplementation decreased the TBI-induced enhancement of these factors (*p* < 0.05) (Fig. 2c).

### ω-3 PUFA supplementation inhibits HMGB1 expression in lesioned cortices

**Fig. 3:**
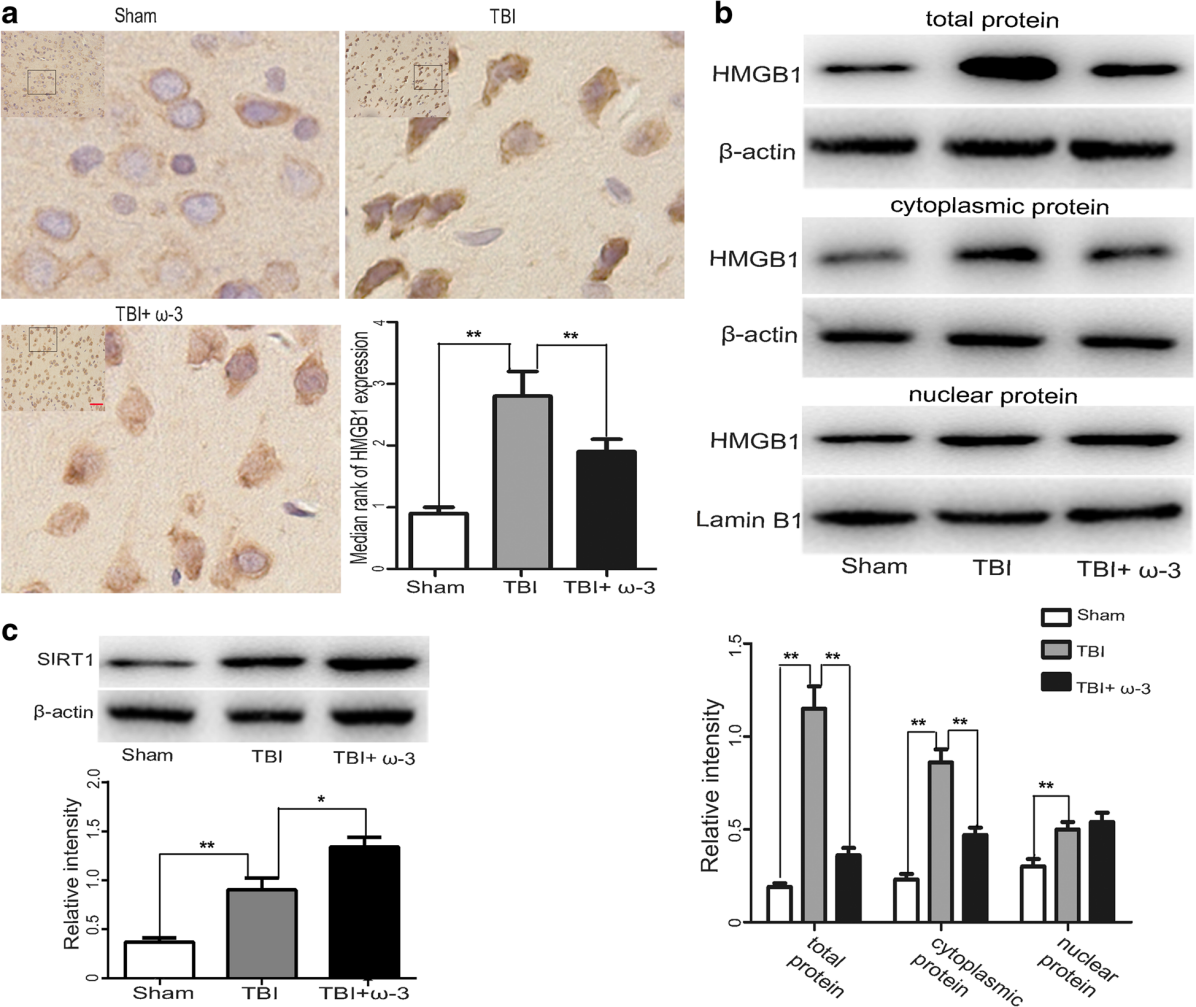
ω-3 PUFA supplementation inhibits HMGB1 expression in lesioned cortices. **a** HMGB1 immunoreactivity in the cortex was significantly decreased by ω-3 PUFA supplementation (1.92 ± 0.22 vs. 2.86 ± 0.39, *p* < 0.05). **b** ω-3 PUFA supplementation significantly decreased HMGB1 expression in cytosolic and total cellular protein levels (*p* < 0.05), but not nuclear protein (*p* > 0.05). **c** SIRT1 protein level was upregulated after ω-3 PUFA supplementation; *n* = 6 in each group. The values are expressed as mean ± standard deviation: *p < 0.05, **p < 0.01, *scale bars* = 50 μm

HMGB1 translocation and release play important roles in TBI-induced microglial activation and the subsequent inflammatory response [10]. As results from the current study showed that ω-3 PUFA supplementation inhibited HMGB1 release, it was necessary to study whether ω-3 PUFA supplementation had the same effect on the expression and translocation of HMGB1. As translocation and release of HMGB1 are known to be closely related to its acetylation levels [36, 37], sirtuins (SIRTs), a family of deacetylases, were also examined to elucidate ω-3 PUFA-mediated inhibition of HMGB1. Western blot analysis demonstrated that expression levels of HMGB1 in the cytosol, nuclei, and in total protein levels of cells from lesioned cortices increased after TBI, but following ω-3 PUFA supplementation, immunohistochemical staining showed decreased expression of HMGB1 3 days after TBI (1.92 ± 0.22 vs. 2.86 ± 0.39, *p* < 0.05) (Fig. 3a). Compared with the TBI group, ω-3 PUFA supplementation effectively decreased HMGB1 expression in the cytosol and in total protein of cells from lesioned cortices (*p* < 0.05), but not in nuclear protein (*p* > 0.05) (Fig. 3b). Finally, SIRT1 protein levels were also upregulated after ω-3 PUFA supplementation (*p* < 0.05) (Fig. 3c).

### ω-3 PUFA supplementation provides neuroprotection via inhibition of HMGB1

**Fig. 4:**
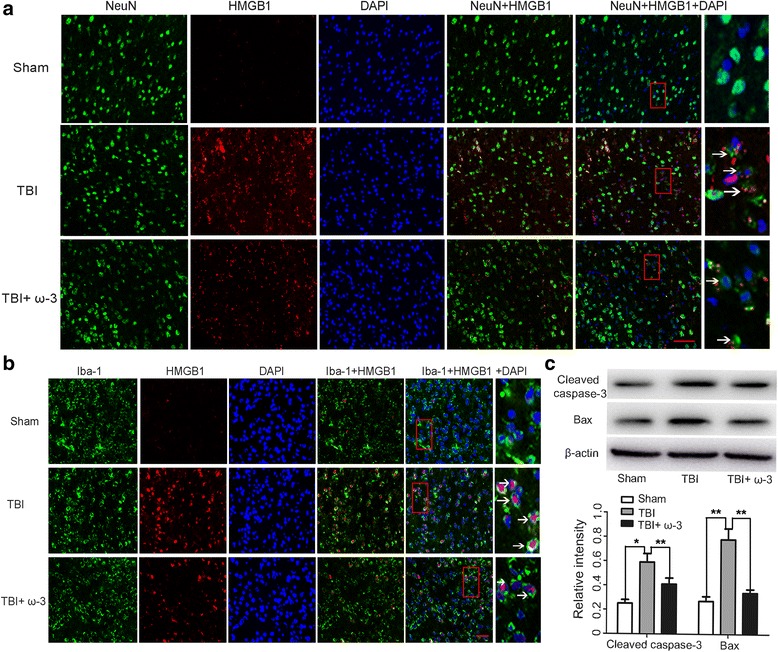
ω-3 PUFA supplementation inhibits HMGB1 expression in lesioned cortices. **a** TBI enhanced the expression of HMGB1 in neurons (NeuN^+^), which was significantly decreased by ω-3 PUFA supplementation (*p* < 0.05). *Arrows* point to HMGB1-positive neurons. **b** TBI enhanced the expression of HMGB1 in microglial cells (Iba-1^+^), which was significantly decreased by ω-3 PUFA supplementation (*p* < 0.05). *Arrows* point to HMGB1-positive microglial cells (Iba-1^+^). **c** ω-3 PUFA supplementation decreased the expression of cleaved caspase-3 and Bax in lesioned cortices (*p* < 0.05); *n* = 6 in each group. The values are expressed as mean ± standard deviation: *p < 0.05, **p < 0.01, *scale bars* = 50 μm

**Fig. 5:**
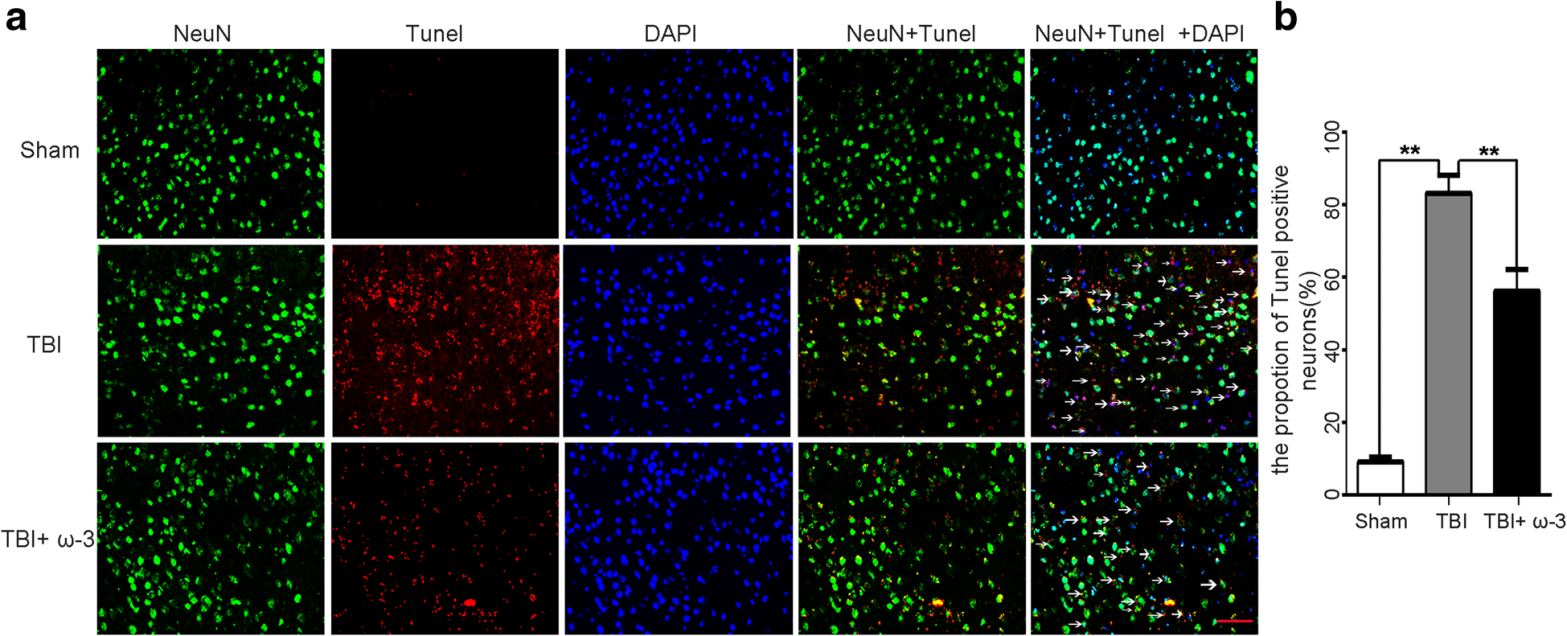
ω-3 PUFA supplementation inhibits neuronal apoptosis in lesioned cortices. **a** ω-3 PUFA supplementation significantly decreased the rate of TUNEL-positive neurons after TBI (56.19 ± 6.60% vs. 83.23 ± 5.46%, *p* < 0.05). *Arrows* point to TUNEL-positive neurons. **b** The rate of neuronal apoptosis in the three groups; *n* = 6 in each group. The values are expressed as mean ± standard deviation: *p < 0.05, ** *p* < 0.01, *scale bars* = 50 μm

Using double immunofluorescent staining, HMGB1 expression in neurons (NeuN^+^) and microglia (Iba-1^+^) was assessed. Compared to the sham group, the expression levels of HMGB1 in both neurons and microglia were higher in the TBI group 3 days after injury. After ω-3 PUFA supplementation, HMGB1 expression was inhibited in both neurons and microglia in lesioned cortices (*p* < 0.05) (Fig. 4a, b). Western blot analysis showed that compared to the TBI group, expression levels of cleaved caspase-3 and Bax were significantly reduced in the TBI + ω-3 group 3 days after TBI (*p* < 0.05) (Fig. 4c). TUNEL staining further demonstrated that TUNEL-positive neurons were significantly decreased in the TBI + ω-3 group 3 days after TBI compared in the TBI group (56.19 ± 6.60% vs. 83.23 ± 5.46%, *p* < 0.05) (Fig. 5). The results suggested that HMGB1 plays an important role in TBI-induced microglial activation and neuronal damage and that ω-3 PUFA supplementation inhibited microglial activation and neuronal apoptosis thus providing neuroprotection through the inhibition of both HMGB1 expression and activity.

### ω-3 PUFA supplementation influences HMGB1-mediated activation of the TLR4/NF-κB signaling pathway in lesioned cortices

**Fig. 6:**
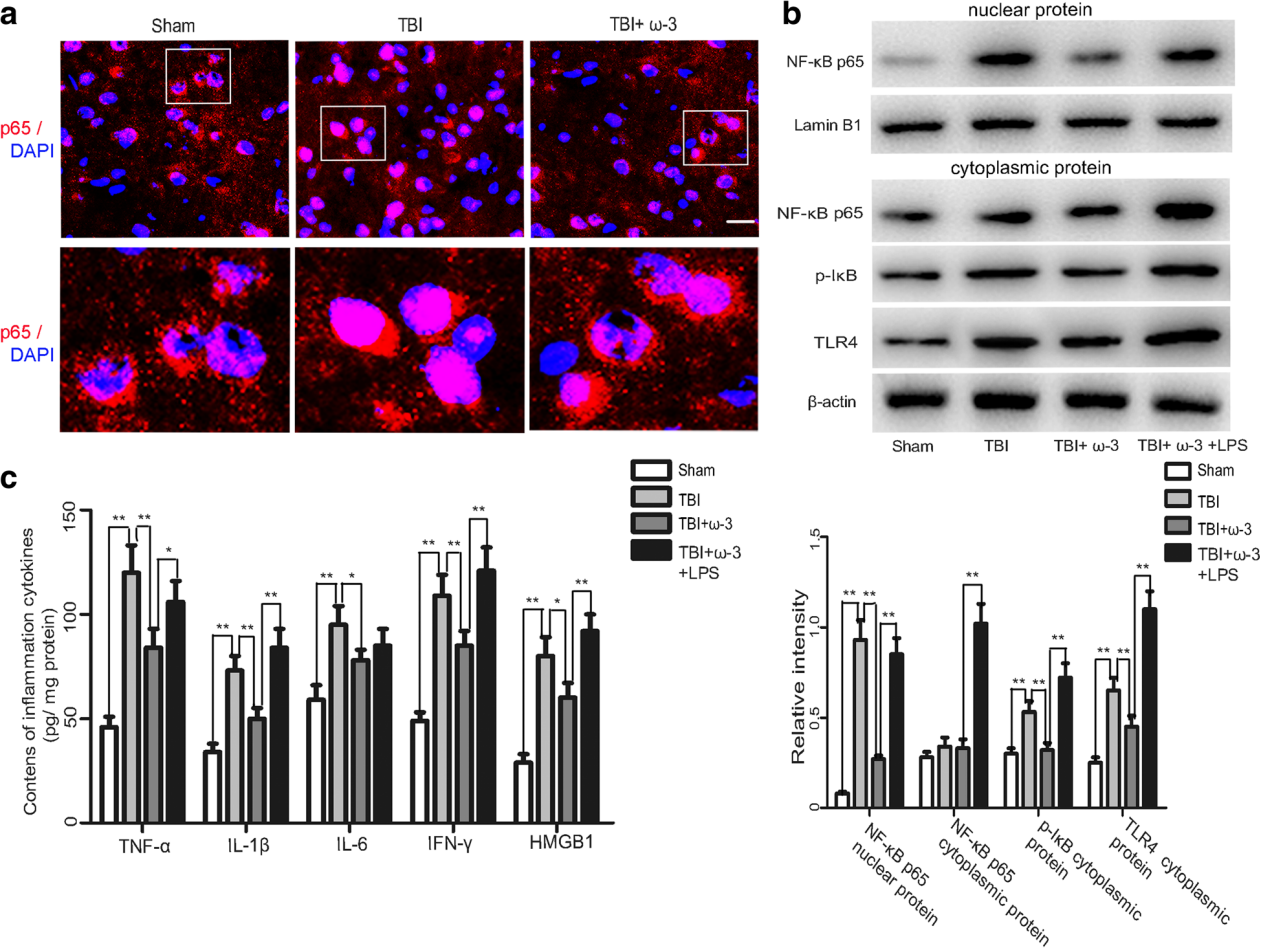
ω-3 PUFA supplementation inhibits HMGB1-mediated activation of the TLR4/NF-κB pathway in lesioned cortices. **a** ω-3 PUFA supplementation inhibited NF-κB p65 translocation to the nucleus (*p* < 0.05). Representative photomicrographs of HMGB1 staining in the experimental groups. **b** The expression of HMGB1-mediated TLR4/NF-κB-related factors (NF-κB p65, p-IκB, and TLR4) increased 3 days after TBI (*p* < 0.05). Subsequent ω-3 PUFA supplementation inhibited NF-κB p65 nuclear translocation and decreased the expression of NF-κB p65, p-IκB, and TLR4. The inhibitory effect of ω-3 PUFA supplementation on the TLR4/NF-κB pathway was reversed by the TLR4 agonist, LPS (*p* < 0.05). **c** The inhibitory effect of ω-3 PUFA supplementation on the neuroinflammatory response was also reversed by LPS (*p* < 0.05); *n* = 6 in each group. The values are expressed as mean ± standard deviation: *p < 0.05, **p < 0.01, *scale bars* = 50 μm

As HMGB1 has been shown to activate the TLR4/NF-κB signaling pathway which induces the release of various inflammatory factors [14], changes in TLR4/NF-κB signaling pathway-related factors (NF-κB p65, p-IκB, and TLR4) were measured after ω-3 PUFA supplementation using western blot analysis and immunofluorescence staining. Data showed that compared to the TBI group, ω-3 PUFA supplementation inhibited NF-κB p65 nuclear translocation and decreased NF-κB p65 expression 3 days after injury (*p* < 0.05). Moreover, ω-3 PUFA supplementation decreased the expression of TLR4/NF-κB-related factors, including p-IκB and TLR4 (*p* < 0.05) (Fig. 6a, b). LPS activates TLR4 signaling to mediate inflammatory responses [11, 38]. As expected, the inhibitory effects of ω-3 PUFA supplementation on the inflammatory response and TLR4/NF-κB pathway were reversed by the TLR4 agonist, LPS (*p* < 0.05) (Fig. 6a, c). Overall, our results indicated that ω-3 PUFA supplementation could inhibit HMGB1-mediated activation of the TLR4/NF-κB pathway and attenuate the subsequent inflammatory response after TBI.

## Discussion

**Fig. 7:**
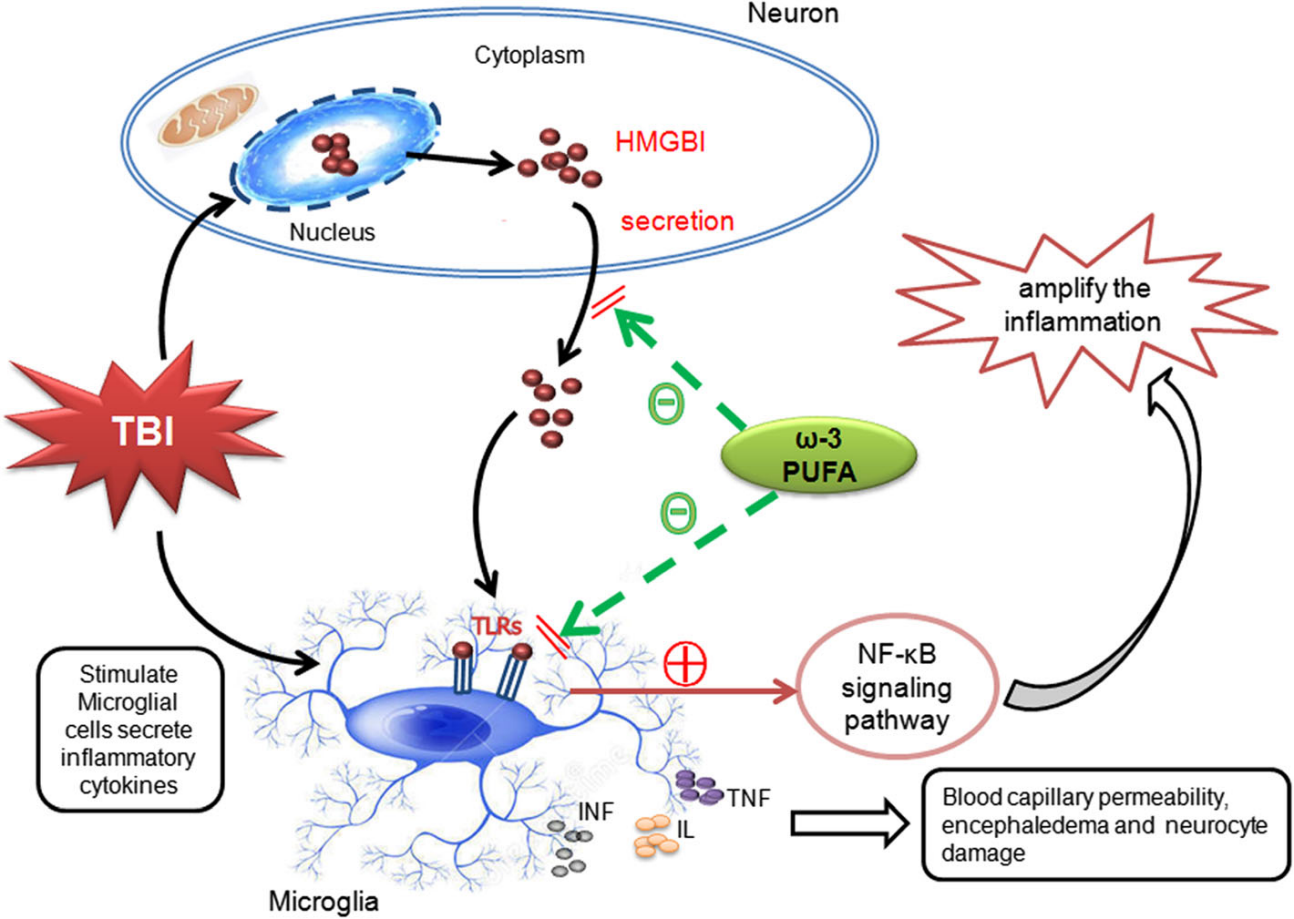
Schematic illustrating the possible neuroprotective mechanisms of ω-3 PUFA supplementation after TBI. As illustrated, TBI-induced microglial activation initiates a neuron-glia neuroinflammatory response by producing a wide array of proinflammatory factors or mediators such as TNF, IL, and IFN. Under inflammatory conditions, elevated HMGB1 binds to transmembrane toll-like receptors (TLRs) to induce further release of inflammatory cytokines. Supplementation with ω-3 PUFA inhibited TBI-induced microglial activation and the subsequent inflammatory response and also facilitated neuronal recovery through inhibition of HMGB1 translocation and release, and HMGB1-mediated activation of the TLR4/NF-κB signaling pathway

In the present study, we demonstrated that ω-3 PUFA, a commonly used clinical immunonutrient, has neuroprotective effects on TBI-induced damage. Based on the Feeney DM TBI model in rats, we showed that ω-3 PUFA supplementation reduced brain edema and improved neurological scores. Data from Nissl staining and western blot analysis also demonstrated that ω-3 PUFA supplementation protected neuron and improved neurological functions by inhibiting TBI-induced expression of the apoptotic factors, cleaved caspase-3, and Bax (Fig. 7).

It has been well established that ω-3 PUFA is the precursor for several components of the neuronal membrane and also that it is essential for neurodevelopment [23–26]. Administration of ω-3 PUFA after injury has been shown to improve neurological outcomes in experimental TBI. The mechanisms involved in this improvement are most likely associated with decreased oxidative stress and neurotrophic support and inhibition of neuron apoptotic pathways [24]. In addition, ω-3 PUFA has antioxidative and anti-inflammatory effects that influence the pathogenesis of many diseases [23–26]. Microglial activation and the subsequent neuroinflammatory response play important roles in secondary injury after TBI [4, 6]. The antioxidative and anti-inflammatory effects of ω-3 PUFA-mediated regulation of inflammation and the release of immune factors has been documented [24, 25]. However, its effects on TBI-induced microglial activation and neuroinflammation have yet to be investigated. Our study showed that activation of microglia and expression of inflammatory factors (TNF-α, IL-1β, IL-6, and IFN-γ) were significantly enhanced in brain tissues after TBI, which were associated with brain edema and neurological deficits. After ω-3 PUFA supplementation, microglial activation was inhibited and there was a decrease in TBI-induced inflammatory factors, suggesting ω-3 PUFA supplementation could decrease microglial activation and the subsequent inflammatory response, reduce brain edema, inhibit apoptosis, and improve neurological functions.

HMGB1 is considered to be the central component of the late inflammatory response. The translocation and secretion of HMGB1 are important step in HMGB1-induced inflammation [7, 39]. After release, HMGB1 binds to transmembrane TLR4 and activates the TLR4/NF-κB signaling pathway, ultimately leading to neuroinflammation in the CNS [9, 10]. The effects of ω-3 PUFA supplementation on HMGB1 translocation and release and HMGB1-induced TLR4/NF-κB activation have, to date, not been fully elucidated. Here, we showed that HMGB1 expression in neurons and microglial cells located in the lesioned cortices was significantly increased after TBI and that ω-3 PUFA supplementation reduced this expression, suggesting that HMGB1 played a critical role in neuroinflammation and neuronal injury after TBI. Moreover, ω-3 PUFA supplementation inhibited the translocation and release of HMGB1 and its subsequent activities after TBI. TUNEL staining and western blot analysis further demonstrated that ω-3 PUFA supplementation inhibited neuronal apoptosis. Our results suggested that HMGB1 has important roles in microglial activation and neuronal apoptosis and that ω-3 PUFA supplementation provides neuroprotection by inhibiting HMGB1 expression and release (Fig. 7).

HMGB1 release is closely related to its acetylation level, which is regulated by protein deacetylase [36, 37]. SIRTs are a family of deacetylases that require nicotinamide adenine dinucleotide (NAD^+^) as a cofactor for the deacetylation reaction [33]. Interestingly, ω-3 PUFA supplementation has been shown to protect against obesity-associated inflammation and colon carcinogenesis through upregulation of SIRT1 [40]. In this study, we also found that SIRT1 protein levels were upregulated after ω-3 PUFA supplementation, indicating that ω-3 PUFA inhibited the expression, translocation, and release of HMGB1 in a SIRT1 deacetylation-mediated-dependent manner. Future studies involving the expression of acetylated HMGB1 and SIRT1 activity should be examined to elucidate the novel anti-inflammatory mechanisms of omega-3 PUFA-mediated inhibition of HMGB1.

In order to further understand the mechanism by which ω-3 PUFA supplementation inhibits HMGB1 expression and TBI-induced inflammation, we investigated the changes in factors involved in the TLR4/NF-κB signaling pathway, which were involved in inflammation processes after ω-3 PUFA supplementation. TLR4, p65, and IκB are known to be major components in the TLR4/NF-κB signaling pathway [36, 37]. Following TLR4 activation by extracellular inflammatory factors, phosphorylation and subsequent release of IκB occurs, leading to activation of NF-κB. Nuclear translocation of NF-κB then induces a cascade of inflammatory responses [36, 37]. Results from the present study showed that ω-3 PUFA supplementation inhibited the translocation of NF-κB p65 from the cytosol to the nucleus, reduced NF-κB p65 expression, and inhibited the expression of the TLR4/NF-κB signaling pathway-associated proteins, p-IκB and TLR4. The inhibitory effects of ω-3 PUFA supplementation on the inflammatory response and on TLR4/NF-κB signaling pathway activation was reversed by LPS, suggesting HMGB1-mediated activation of the TLR4/NF-κB pathway has an important role in TBI-induced inflammatory responses. Future studies involving *HMGB1* or *TLR4* knockout mice are warranted to further investigate the mechanisms involved in ω-3 PUFA-mediated inhibition of HMGB1 and subsequent activation of the TLR4/NF-κB pathway. In addition, other transmembrane receptors for HMGB1, such as RAGE, should be investigated [41] to facilitate the understanding of the mechanisms involved in the anti-inflammatory effects of ω-3 PUFA supplementation.

## Conclusions

Taken together, our study showed that the immunonutrient, ω-3 PUFA, inhibited HMGB1 translocation and extracellular release, thus affecting HMGB1-mediated activation of the TLR4/NF-κB signaling pathway. Ultimately, this led to a reduction in TBI-induced microglial activation and the subsequent inflammatory response thus providing neuroprotection.

## Abbreviations

ELISA: Enzyme-linked immunosorbent assay
HMGB1: High-mobility group box 1
IFN: Interferon
IL: Interleukin
LPS: Lipopolysaccharide
mNSS: Modified neurological severity scores
NAD: Nicotinamide adenine dinucleotide
NF-κB: Nuclear factor-κB
RAGE: Receptor for advanced glycation end products
TBI: Traumatic brain injury
TLRs: Toll-like receptors
TNF: Tumor necrosis factor
ω-3 PUFA: Omega-3 polyunsaturated fatty acid
